# Safety and tolerability of asunercept plus standard radiotherapy/temozolomide in Asian patients with newly-diagnosed glioblastoma: a phase I study

**DOI:** 10.1038/s41598-021-02527-1

**Published:** 2021-12-15

**Authors:** Kuo-Chen Wei, Peng-Wei Hsu, Hong-Chieh Tsai, Ya-Jui Lin, Ko-Ting Chen, Cheng-Hong Toh, Hui-Lin Huang, Shih-Ming Jung, Chen-Kan Tseng, Yu-Xiong Ke

**Affiliations:** 1 Department of Neurosurgery, Chang Gung Memorial Hospital, Linkou, 5 Fuxing St., Guishan Dist., Taoyuan, 33305 Taiwan; 2 School of Medicine, Chang Gung University, 259 Wenhua 1st Rd., Guishan Dist., Taoyuan, 33302 Taiwan; 3 Graduate Institute of Clinical Medical Sciences and School of Traditional Chinese Medicine, Chang Gung University, 259 Wenhua 1st Rd., Guishan Dist., Taoyuan, 33302 Taiwan; 4 Department of Medical Imaging and Intervention, Chang Gung Memorial Hospital, Linkou, Taoyuan, Taiwan; 5 Clinical Trial Center, Chang Gung Memorial Hospital, Linkou, Taoyuan, Taiwan; 6 Department of Pathology, Chang Gung Memorial Hospital, Linkou, 5 Fuxing St., Guishan Dist., Taoyuan, 33305 Taiwan; 7 Department of Radiation Oncology, Chang Gung Memorial Hospital at Linkou, Chang Gung University, Taoyuan, Taiwan; 8 CANbridge Pharmaceuticals Inc., Shanghai, China

**Keywords:** Cancer, Drug discovery

## Abstract

Asunercept (company code APG101 [Apogenix AG]; company code CAN008 [CANbridge Pharmaceuticals]) is a novel glycosylated fusion protein that has shown promising effectiveness in glioblastoma. This Phase I study was initiated to evaluate the tolerability and safety of asunercept in combination with standard radiotherapy and temozolomide (RT/TMZ) in Asian patients with newly diagnosed glioblastoma. This was the Phase I portion of a Phase I/II open label, multicenter trial of asunercept plus standard RT/TMZ. Adults with newly-diagnosed glioblastoma received surgical resection followed by standard RT/TMZ plus asunercept 200 mg/week (Cohort 1) or 400 mg/week (Cohort 2) by 30-min IV infusion. The primary endpoint was the safety and tolerability of asunercept during concurrent asunercept and RT/TMZ; dose-limiting toxicities were observed for each dose. Secondary endpoints included pharmacokinetics (PK) and 6-month progression-free survival (PFS6). All patients (Cohort 1, n = 3; Cohort 2, n = 7) completed ≥ 7 weeks of asunercept treatment. No DLTs were experienced. Only one possibly treatment-related treatment emergent adverse event (TEAE), Grade 1 gingival swelling, was observed. No Grade > 3 TEAEs were reported and no TEAE led to treatment discontinuation. Systemic asunercept exposure increased proportionally with dose and showed low inter-patient variability. The PFS6 rate was 33.3% and 57.1% for patients in Cohort 1 and 2, respectively. Patients in Cohort 2 maintained a PFS rate of 57.1% at Month 12. Adding asunercept to standard RT/TMZ was safe and well tolerated in patients with newly-diagnosed glioblastoma and 400 mg/week resulted in encouraging efficacy.

**Trial registration** NCT02853565, August 3, 2016.

## Introduction

Glioblastoma is the most common and aggressive malignant tumor of the central nervous system, with an estimated 5-year survival of 5.5%^1^. New therapeutic options are needed and an active area of research is the CD95/Fas receptor and its ligand CD95L/FasL, which are involved in tumor progression, invasiveness, the development of resistance to radiotherapy and immune therapy and survival of cancer stem cells after therapy, which is associated with relapse^2^. Preclinical studies have shown that CD95L from autocrine and paracrine sources contributes to an invasive phenotype of glioblastoma cells and neutralisation of CD95 reduces the invasive migration of glioblastoma cells^3,4^. Further in vitro data indicate that blocking the activation of CD95 by CD95L reduces the increased invasiveness of irradiated glioblastoma cells that occurs in response to radiation^5,6^. Preventing glioblastoma cell invasion into the surrounding normal brain tissue by blocking the CD95–CD95L pathway is therefore hypothesized to enhance the clinical effectiveness of both radiation therapy and surgery. CD95L has also been associated with an immunosuppressive tumour microenvironment, and CD95L inhibition has been shown to suppress programmed cell death of immune T cells^7^.

Asunercept (APG101, CAN008) is a first-in-class recombinant glycosylated fusion protein consisting of the extracellular domain of human CD95 (APO-1/Fas) and the Fc domain of human IgG1, designed to selectively bind to CD95L and disrupt CD95/CD95L signaling^8^. In a Phase I study including 34 healthy volunteers, four and five consecutive intravenous infusions of asunercept 400 mg/week led to comparable maximum plasma concentrations (C_max_) at steady state (304.0 μg/mL and 224.97 μg/mL, respectively), with a terminal half-life of 12.0–15.5 days.(13) Based on the results of this study, asunercept 400 mg/week was identified as a clinically relevant dose^8^.

A subsequent proof-of-concept Phase II study in patients with glioblastoma at first or second progression showed the addition of asunercept to radiotherapy is well-tolerated and provides clinical benefit; 6-month progression-free survival (PFS-6; the primary endpoint) was 3.8% (95% confidence interval [CI] 0.1–19.6) for radiotherapy compared with 20.7% (95% CI 11.2–33.4) for radiotherapy plus asunercept (p = 0.048)^9^. In addition, in patients with recurrent glioblastoma, the addition of asunercept to reirradiation is associated with prolonged time to deterioration and maintenance of quality of life beyond disease progression compared with reirradiation alone^10^. Reflecting the current unmet need for effective treatments for glioblastoma, the U.S. Food and Drug Administration and the European Medicines Agency (EMA) have assigned asunercept orphan drug status for the treatment of glioblastoma or glioma, respectively^11,12^. It has also received PRIME designation by the EMA for the treatment of glioblastoma^13^.

Asunercept is currently under evaluation in an on-going clinical trial in patients with newly-diagnosed glioblastoma in combination with the current standard treatment of surgical resection followed by radiotherapy concomitant with the oral alkylating agent temozolomide (RT/TMZ), and then six maintenance cycles of temozolomide delivered over 6 months^14–17^. However, data are not yet available. To the authors’ knowledge, the pharmacokinetics (PK) of asunercept have not been evaluated in newly diagnosed glioblastoma when combined with standard therapy. Our Phase I study described herein was therefore initiated to evaluate the tolerability and safety of asunercept in combination with standard RT/TMZ in Asian patients with newly diagnosed glioblastoma. This study also sought to assess 400 mg as a safe dose for use in the Phase II component of this trial, to evaluate the PK of asunercept, and to provide a preliminary assessment of clinical efficacy.

## Methods

### Study design and patients

This was a Phase I component of a Phase I/II open label, multicenter trial consisting of a screening phase of approximately 4 weeks, a 7-week chemoradiotherapy treatment with standard RT/TMZ and concomitant asunercept, and an adjuvant treatment phase consisting of concomitant asunercept and temozolomide. The study was conducted at three hospitals in Taiwan in accordance with the principles of the Declaration of Helsinki and International Council for Harmonization Good Clinical Practice. The trial protocol was registered at clinicaltrials.gov on August 3, 2016 (NCT02853565).

The study included adult Taiwanese patients (aged ≥ 20 to < 75 years) with newly diagnosed and histologically confirmed glioblastoma, life expectancy ≥ 6 months, and a tumor that was surgically accessible with available tissue samples. Eligible patients also had a Karnofsky Performance Scale (KPS) Index score ≥ 60 prior to treatment and had not received prior therapy for brain tumors. Detailed patient enrollment criteria are provided in Online Resource 1.

### Study treatments

All eligible patients received a backbone of standard RT/TMZ therapy. In total, patients received approximately 60 Gy radiation therapy; 2 Gy/day from Monday to Friday (10 Gy/week), for a total of 30 radiation treatments (approximately 6 weeks). From the first day of radiotherapy until the end of radiotherapy, temozolomide (Temodar, Merck, USA) 75 mg/m^2^/day was administered orally once daily for a maximum of 7 weeks. All patients then had 4 weeks off treatment before initiating a maintenance treatment phase consisting of temozolomide 150 mg/m^2^/day on Days 1 to 5 of the first 28-day cycle, and 200 mg/m^2^/day on Days 1 to 5 of five additional 28-day cycles.

In addition to the standard treatment backbone, patients were assigned sequentially to receive asunercept (APG101, Apogenix AG, Germany) 200 mg/week (Cohort 1) or 400 mg/week (Cohort 2). Asunercept was administered as a 30-min intravenous infusion, concurrently with RT/TMZ, and then in combination with temozolomide during the maintenance phase, until disease progression or unacceptable toxicity. For each cohort, there was an interval of at least 2 weeks between the first and second patient enrolled. The doses and dose schedule for asunercept used in this Phase I study were selected based on results of previous Phase I and II studies^8,9^.

### Asunercept cohort patient recruitment

Planned enrolment included three to six patients to Cohort 1, and three to nine patients to Cohort 2, depending on the observed safety of asunercept, in accordance with the algorithm described in Online Resource 1.

### Endpoints and measurements

The primary endpoint of this study was evaluation of the safety and tolerability of asunercept. Secondary endpoints were determination of the recommended asunercept dose for use in the Phase II component of the study (based on observed toxicities, the overall safety profile of asunercept, and results from a previous Phase II study of asunercept^9^), analysis of the PK profile of asunercept and primary assessment of PFS and PFS-6.

A full schedule of assessments is given in Online Resource 1. In brief, tumor response was assessed by MRI using the Response Assessment in Neuro-Oncology (RANO) criteria^18^. Baseline or pre-study MRI imaging was conducted within 2 days of surgery, and radiotherapy planning MRI was accepted as baseline images if the imaging parameters were identical to the follow-up MRIs. Subsequent follow-up brain MRI was carried out every 8 weeks (± 5 days) following radiotherapy. Survival assessments began post-treatment at the 28-day follow-up visit and were then conducted every 8 weeks.

A DLT was defined as any observed toxicity deemed to be study drug-related occurring during the initial 6-week concurrent use of asunercept with RT/TMZ. This included Grade > 2 hematological toxicities present for > 4 days, Grade > 2 non-hematological toxicities, and any other toxicity increasing in severity from baseline that was deemed clinically significant and/or unacceptable by the investigator and after review by the SMC, or that resulted in a disruption of the dosing schedule for > 14 days. The severity of adverse events (AEs) was graded according to National Cancer Institute (NCI) Common Terminology Criteria for Adverse Events (CTCAE) Version 4.03.

PFS was defined as the time from the date of the first dose of asunercept to the date of the first radiologically documented disease progression (per local investigator assessment according to RANO criteria) or death due to any cause. PFS-6 was defined as the crude rate of patients free of disease progression at 6 months after the first dose of asunercept.

### Pharmacokinetic analysis

Blood for PK analysis was collected at the first treatment visit and then weekly during Weeks 2–6. Approximately 2 mL of peripheral blood was drawn from the contralateral arm to the asunercept infusion < 5 min before the start of each infusion and following the first dose at 5 min (± 2 min), 30 min (± 5 min), 1 h (± 5 min), 2 h (± 5 min), 4 h (± 5 min), and 24 h (± 5 min). Additional blood samples were taken weekly before asunercept administration during the chemoradiotherapy treatment block. Blood samples were collected a total of 12 times for each patient at each study center and were sent for analysis at a central laboratory (Covance, Taiwan). All PK parameters were calculated using Phoenix WinNonlin (Certara, Version 8.1) with a non-compartmental method.

### Biomarker analysis

This study included an exploratory retrospective investigation of the biomarkers O6-methylguanine methyltransferase (MGMT methylation status), CD95L expression and methylation of a single cytosine-phosphate-guanine site (CpG2) upstream of the CD95L promoter, for their predictive/prognostic value. These biomarkers were investigated in the proof-of-concept Phase II study in patients with glioblastoma at first or second progression. For each biomarker, pre-existing tumor samples from surgery were collected, stored, and sent to a central laboratory for analysis.

MGMT promoter methylation status was determined by pyrosequencing. The mean methylation ratio cutoff value ≥ 10% was defined to classify MGMT methylated vs. unmethylated cases^19^. CD95L expression in tumor cells was assessed by immunohistochemistry (IHC). Positive CD95L expression was defined as 2 + or 3 + staining, and 0 or 1 + was considered negative. Unlike previous studies, which used pyrosequencing to detect CpG2 methylation, the present study used real-time fluorescent quantitative polymerase chain reaction (q-PCR). Full details of the CpG2 methylation detection methodology are provided in Online Resource 1. The threshold for CpG2 methylation was defined as 52% (‘High’ methylation ≥ 52% and ‘low’ < 52%) based on the median values from a currently unpublished study and confirmation in a validation set of 70 patients from a study conducted in China and Taiwan (Online Resource 2: Supplementary Table 1).

### Statistics

Patients were considered evaluable if they completed 6 weeks of concurrent treatment or experienced any DLT. Patients who dropped out for reasons other than safety during the DLT evaluation period could be replaced. The safety population included all patients who received ≥ 1 dose of asunercept. The PK analysis population included all patients who received ≥ 1 dose of asunercept and for whom PK parameters were calculated. The full analysis set included all patients who received ≥ 1 dose of asunercept and underwent ≥ 1 tumor MRI session following the first dose of asunercept.

For time-to-event variables, Kaplan–Meier plots and median time-to-event are presented. For the PFS-6 rate, a two-sided 95% CI was calculated using the exact method (Clopper–Pearson). Missing safety data were not imputed, except for handling of partial dates for AEs. Missing efficacy data were not imputed and were regarded as non-response or censored. All statistical analyses were performed using SAS®, Version 9.2 or higher.

### Ethics approval

The study protocol was approved by local ethics review boards (Chang Gung Memorial Hospital, Linkou: Chang Gung Medical Foundation Institutional Review Board [201600387A0C604]; National Taiwan University Hospital: Research Ethics Committee B, National Taiwan University Hospital [201603103MSB]; Tri-Service General Hospital: Institutional Review Board, Tri-Service General Hospital [1-106-01-007]).

### Consent to participate

All patients provided written informed consent before entering the study.

## Results

### Patients

A total of 12 patients with newly diagnosed glioblastoma were screened and ten patients were assigned to treatment between September 9, 2016 and September 28, 2018 (three patients to Cohort 1 and seven patients to Cohort 2). All ten patients received ≥ 1 dose of asunercept, completed ≥ 7 weeks of treatment, and completed the study per protocol (or were still receiving treatment at the cut-off date; 28 September, 2018). There were no major protocol deviations leading to exclusion of patients from the planned analysis populations. By October 14, 2019 all patients had stopped treatment, and had received treatment for a range of 66–106 weeks. Patient demographics and baseline characteristics are summarised in Table 1. All patients underwent surgical procedures, with the majority (70%) undergoing total resection.

**Table 1:** Patient demographics and baseline characteristics.

Variable^a^	Cohort 1, 200 mg/week (n = 3)	Cohort 2, 400 mg/week (n = 7)	Total (N = 10)
**Age, years**			
Mean (SD)	55.0 (7.6)	51.7 (12.6)	52.7 (11.0)
Median (min, max)	56.0 (47.0, 62.0)	52.0 (34.0, 73.0)	54.0 (34.0, 73.0)
Males, n (%)	2 (66.7)	6 (85.7)	8 (80.0)
Height, cm	166.3 (5.0)	169.2 (9.3)	168.3 (8.1)
Weight, kg	68.6 (15.0)	67.0 (9.4)	67.5 (10.5)
BMI, kg/m^2^	24.7 (3.9)	23.4 (2.7)	23.8 (2.9)
**Karnofsky Performance Index score, n (%)**			
50–70	1 (33.3)	0	1 (10.0)
80–100	2 (66.7)	7 (100.0)	9 (90.0)
**Time since histopathological diagnosis, months**			
Mean (SD)	0.46 (0.36)	0.60 (0.24)	0.56 (0.27)
Median (min, max)	0.46 (0.10, 0.82)	0.66 (0.16, 0.82)	0.64 (0.10, 0.82)
**Surgical procedure, n (%)**			
Total resection	2 (66.7)	5 (71.4)	7 (70.0)
Partial resection	1 (33.3)	2 (28.6)	3 (30.0)
Concomitant medications	3 (100.0)	7 (100.0)	10 (100)
**Concomitant medications received by ≥ 30% of total patients, n (%)**			
Ondansetron	3 (100.0)	7 (100.0)	10 (100.0)
Magnesium oxide	1 (33.3)	6 (85.7)	7 (70.0)
Paracetamol	2 (66.7)	3 (42.9)	5 (50.0)
Sennoside a + b	2 (66.7)	3 (42.9)	5 (50.0)
Valproate sodium	3 (100.0)	2 (28.6)	5 (50.0)
Dexamethasone	1 (33.3)	2 (28.6)	3 (30.0)
Famotidine	0 (0)	3 (42.9)	3 (30.0)
Mannitol	2 (66.7)	1 (14.3)	3 (30.0)

^a^Mean (SD) unless otherwise stated.

### Safety and tolerability

Asunercept medication exposure is summarised in Online Resource 2: Supplementary Table 2. Three patients had TMZ dose interruptions (30%) including one patient in Cohort 1 who missed one dose of TMZ due to poor compliance. No patient in the study experienced a DLT and asunercept 400 mg/week was determined to be the appropriate dose for use in the Phase II component of this study. Among all patients, the most common treatment emergent AEs (TEAEs) were alopecia (60%) and constipation (60%) (Table 2). The most frequent system organ classes affected by TEAEs were skin and subcutaneous tissue disorders (90%), gastrointestinal disorders (60%), and metabolism and nutrition disorders (50%). Treatment-emergent AEs (TEAEs) were reported in all ten patients (a total of 68 events). Two patients in Cohort 2 reported Grade 3 TEAEs that were considered not related to study drugs, and there were no TEAEs greater than Grade 3 or that led to treatment discontinuation (Table 2). Only one TEAE, a Grade 1 gingival swelling, was considered possibly related to asunercept. Two serious AEs were reported in two patients in Cohort 2 (Grade 3 hemorrhoids and Grade 2 seizure).

**Table 2:** Summary of safety findings.

n (%)	Cohort 1, 200 mg/week (n = 3)	Cohort 2, 400 mg/week (n = 7)	Total (N = 10)
Patients experiencing ≥ 1 TEAE	3 (100.0)	7 (100.0)	10 (100.0)
Total number of TEAEs, n	20	48	68
**TEAEs by grade**^**a**^			
Grade 1	0	1 (14.3)^b^	1 (10.0)
Grade 2	3 (100.0)	4 (57.1)	7 (70.0)
Grade 3	0	2 (28.6)	2 (20.0)
**TEAEs occurring in ≥ 20% of total patients**			
Alopecia	2 (66.7)	4 (57.1)	6 (60.0)
Constipation	1 (33.3)	5 (71.4)	6 (60.0)
Dermatitis	1 (33.3)	2 (28.6)	3 (30.0)
Nausea	0	3 (42.9)	3 (30.0)
Hyperglycemia	2 (66.7)	1 (14.3)	3 (30.0)
Urinary tract infection	2 (66.7)	1 (14.3)	3 (30.0)
Decreased appetite	1 (33.3)	1 (14.3)	2 (20.0)
Hiccups	1 (33.3)	1 (14.3)	2 (20.0)
Cough	1 (33.3)	1 (14.3)	2 (20.0)
Dry eye	1 (33.3)	1 (14.3)	2 (20.0)

*TEAE* treatment emergent adverse event.

^a^National Cancer Institute Common Terminology Criteria for Adverse Events (NCI CTCAE).

^b^Gingival swelling considered possibly related to asunercept treatment.

### Asunercept pharmacokinetics

Peak serum asunercept concentrations were generally achieved shortly after the end of the 30 min infusion, with time of the maximum observed serum concentration (T_max_) estimates ranging from 1.0 to 2.0 h for Cohort 1 and 0.58 to 2.5 h for Cohort 2. No notable difference in T_max_ was observed between the 200 and 400 mg/week cohorts (Table 3). Systemic exposure to asunercept, as measured by C_max_ and area under the serum concentration–time curve until 168 h post start of infusion (AUC_0-168_), increased approximately proportionally to dose across the 200 and 400 mg/week dose range, with 2.2- and 2.3-fold increases, respectively. In general, as assessed from the coefficient of variance (CV%), low inter-patient variability was observed for AUC_0-168_ and C_max_. Following repeated weekly dosing, asunercept pre-dose concentrations on Week 6 were on average 3.1- to 3.2-fold higher compared with Week 2 pre-dose levels, with a geometric mean of 57,700 ng/mL versus 18,700 ng/mL for the 200 mg/week dose, and 154,000 ng/mL versus 48,400 ng/mL for the 400 mg/week dose, respectively.

**Table 3:** Summary of asunercept pharmacokinetics.

Variable^a^	AUC_0-tlast_ (µg.h/mL) [CV%]	AUC_0-168_ (µg.h/mL) [CV%]	C_max_ (µg/mL) [CV%]	Median T_max_ (h) [min, max]	Median T_last_ (h) [min, max]
Cohort 1, 200 mg (n = 3)	4910.0 [28.3]	4850.0 [28.1]	57.8 [11.7]	1.50 [1.00, 2.00]	171 [171, 171]
Cohort 2, 400 mg (n = 7)	11,700.0 [17.9]	11,100.0 [18.9]	127.0 [24.6]	1.50 [0.583, 2.50]	172 [169, 196]

*AUC*_*0–168*_ area under the serum concentration–time curve from time zero to 168 h post start of infusion, *AUC*_*0–tlast*_ area under the serum concentration–time curve from time zero to the time of the last measurable concentration, *C*_*max*_ maximum observed serum concentration, *CV%* coefficient of variation, *T*_*last*_ time of the last quantifiable serum concentration, *T*_*max*_ time of the maximum observed serum concentration.

^a^Data are geometric means unless otherwise stated.

### Efficacy

**Figure 1:**
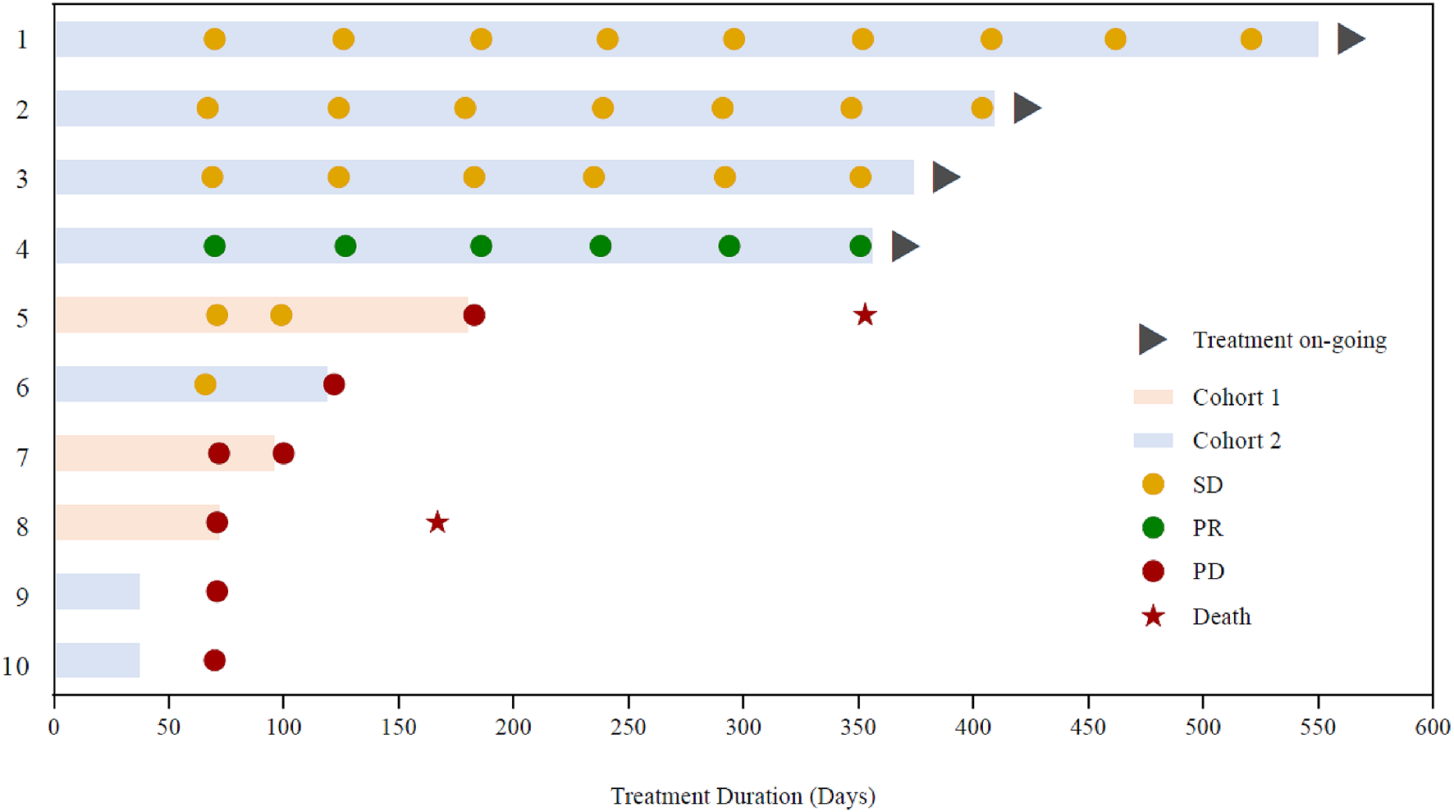
Swimmer plot showing treatment duration, best overall responses and outcomes for each patient.

The PFS-6 rate was 33.3% for patients in Cohort 1 and 57.1% for patients in Cohort 2 (Table 4). The median PFS was 2.4 months for Cohort 1 and was not reached for Cohort 2 owing to four of the seven patients with on-going treatment and no disease progression at the time of cut-off. Of the ten patients included in the analysis, 50% were progression free at 6 months and 40% were progression free at 9 months and 12 months. A swimmer plot showing treatment duration and outcomes for each patient is shown in Fig. 1.

**Table 4:** Summary of progression-free survival (PFS).

	Cohort 1, 200 mg/week (n = 3)	Cohort 2, 400 mg/week (n = 7)	Total (N = 10)
Median PFS^a^, months (95% CI)	2.37 (2.33, 6.01)	NE (2.30, NE)	5.01 (2.30, NE)
Events, n	3	3	6
**Progression-free rates, % (95% CI)**			
3 months	33.3 (0.9, 77.4)	71.4 (25.8, 92.0)	60.0 (25.3, 82.7)
6 months	33.3 (0.9, 77.4)	57.1 (17.2, 83.7)	50.0 (18.4, 75.3)
9 months	0.0	57.1 (17.2, 83.7)	40.0 (12.3, 67.0)
12 months	0.0	57.1 (17.2, 83.7)	40.0 (12.3, 67.0)
PFS-6, % (95% CI)^b^	33.3 [0.8, 90.6]	57.1 [18.4, 90.1]	50.0 [18.7, 81.3]
Disease progression within 6 months, n	2	3	5

*NE* not evaluable (four patients in Cohort 2 had not experienced progression at the time of cut-off).

^a^Kaplan–Meier methodology is used to estimate median time and its 95% confidence interval (CI).

^b^Exact 95% CI is calculated according to Clopper–Pearson.

### Biomarker assessment

No association was observed between MGMT methylation status and treatment outcome. MGMT methylation was detected in five patients (50%), four of whom experienced disease progression before the study cut-off date.

**Figure 2:**
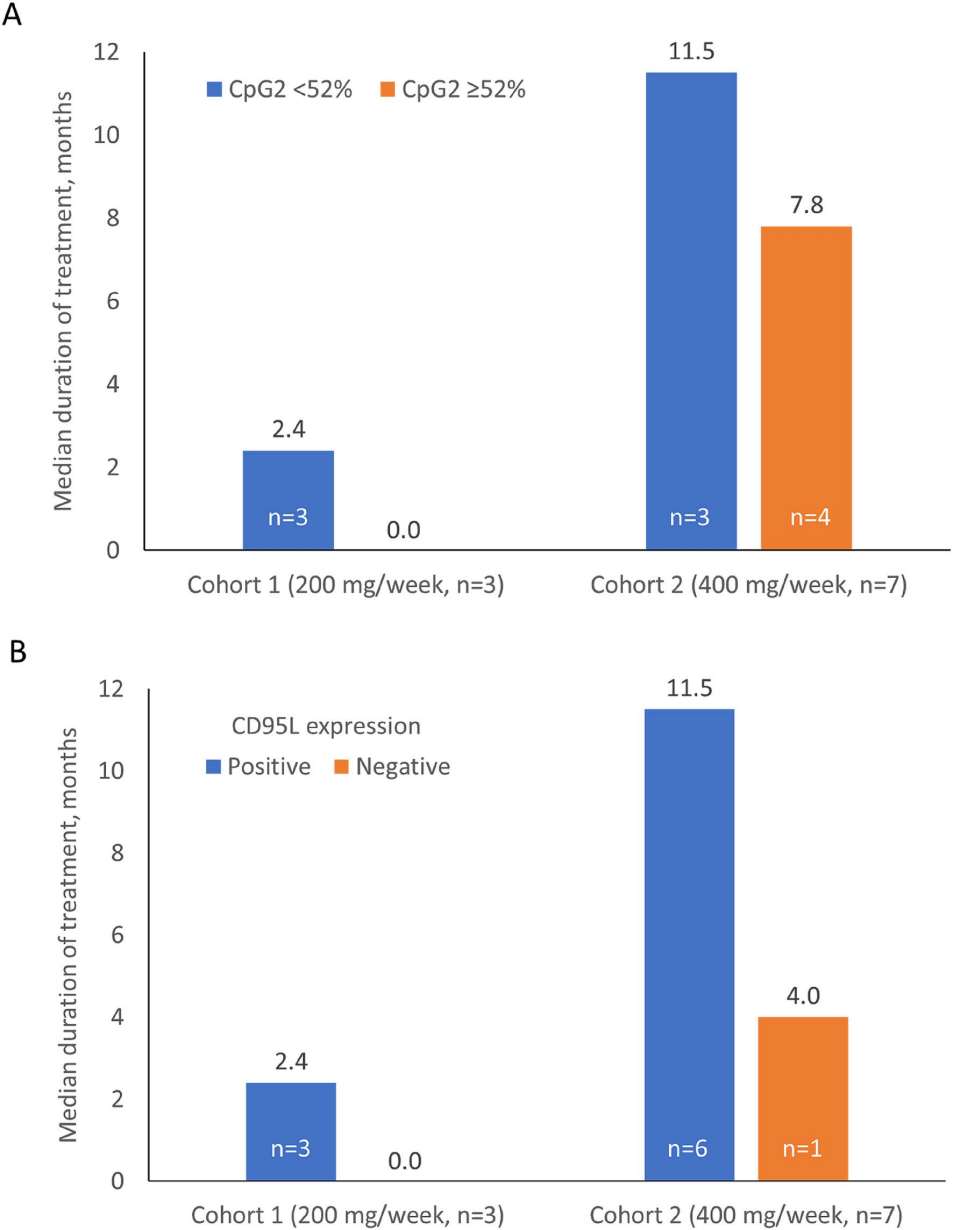
Median duration of treatment by (**A**) CpG2 methylation status (< 52% vs. ≥ 52%) and (**B**) CD95L expression (positive vs. negative).

CpG2 methylation status was assessed in all ten patients. All three patients in Cohort 1 had low (< 52%) CpG2 methylation status (median 42.6%) with a median duration of treatment 2.4 months, and all experienced disease progression (Fig. 2A). Four patients in Cohort 2 had high (median 58.8%), and three had low (median 42.1%) CpG2 methylation. For the patients with high CpG2 methylation, the median duration of treatment was 7.8 months. Conversely, the three patients in Cohort 2 with low CpG2 methylation had a median duration of treatment of 11.5 months. In addition, among patients in Cohort 2, median CpG2 methylation was higher for patients experiencing progressive disease (57.6%) than for those patients who achieved a partial response (42.1%).

Expression of CD95L was positive in nine patients overall and these patients appeared to have a longer duration of treatment, although given the limited sample size this shoud be interpreted with caution (Fig. 2B). In addition, in Cohort 2, the single patient negative for CD95L experienced progressive disease following a treatment duration of 4.0 months. In contrast, among the six patients positive for CD95L, the median duration of treatment was 11.5 months. Four patients who were IHC 3 + positive for CD95L expression had stable or partial response and no disease progression at the time of data cut-off. The remaining patient in Cohort 2 had IHC 2 + positive CD95L expression and experienced progressive disease. Individual CD95L promoter methylation and CD95L expression data are presented in Online Resource 2: Supplementary Table 3.

## Discussion

Data from the present study indicate that asunercept 200 mg/week or 400 mg/week is well tolerated when added to standard RT/TMZ in Asian patients with newly diagnosed glioblastoma. There were no DLTs or Grade > 3 treatment-related TEAEs, and no TEAEs that led to study discontinuation, and therefore 400 mg/week will be used for the Phase II component of this trial. Only one TEAE, a Grade 1 event of gingival swelling, was considered to be possibly related to asunercept. Overall, the safety profile of asunercept in this Phase I trial was in-line with that reported in previous Phase I and Phase II studies of asunercept in glioblastoma^8,9^, with no new safety signals observed. In particular, the proof-of-concept Phase II study in 91 patients with recurrent glioblastoma showed the combination of asunercept and radiotherapy was well tolerated, with no serious AEs causally related to asunercept, and no impairment of radiotherapy tolerability^9^.

The PK parameters for asunercept, as assessed in ten Asian patients with newly-diagnosed glioblastoma receiving standard therapy in this Phase I study, were similar to those reported previously in a PK study in 34 healthy volunteers as well as two Caucasian patients with advanced glioblastoma^8^. In both PK analyses, maximum asunercept serum concentrations were reached shortly after the end of the infusion, with a median T_max_ of 1.5 h observed in the present study. Also in-line with the previous PK analysis^8^, in the present study systemic exposure to asunercept increased approximately proportionally to dose over the range studied and interpatient variability in systemic exposure was low.

Biomarkers are increasingly being used to allocate patients to targeted therapies^20^, although validated biomarkers for patients with glioblastoma have not been identified. DNA methylation is an important regulator of gene expression and may provide a basis for effective glioma diagnosis^20,21^. Previous research in patients with recurrent glioblastoma indicated that low methylation at the CpG2 site within the CD95L promotor was associated with a greater response to asunercept and radiotherapy versus high methylation (hazard ratio 0.19; 95% CI, 0.06–0.58)^9^. Similarly, the present study also found that low CpG2 methylation status appeared to be associated with improved treatment outcomes for patients receiving asunercept 400 mg/week, however, it should be noted that the methods for CpG2 methylation analysis were different in the previous and present study and different thresholds were used. High expression of CD95L (3 + positive) also appeared to be associated with favorable treatment outcomes in this study. These clinical findings are supported by in vitro research that has demonstrated CpG2 methylation is associated with the aggressiveness of glioma-derived cancer spheroids, with the invasive growth of these spheroids suppressed in the presence of asunercept^22^. Therefore, CpG2 methylation of the CD95L promoter represents a potential biomarker for predicting response to therapy with asunercept in patients with glioblastoma.

In conclusion, this Phase I study indicated that adding asunercept to standard RT/TMZ is well tolerated in Asian patients with newly diagnosed glioblastoma. Asunercept 400 mg/week was recommended for use in the ongoing Phase II component of this trial. Encouragingly, patients who received asunercept 400 mg/week maintained a PFS rate of 57.1% between Month 6 and Month 12 of follow-up.

## Supplementary Information

Supplementary Information 1.

Supplementary Information 2.

## Data Availability

The datasets generated during and/or analysed during the current study are available from the corresponding author on reasonable request.
