# Supplementary material for: Safety and tolerability of asunercept plus standard radiotherapy/temozolomide in Asian patients with newly-diagnosed glioblastoma: a phase I study
Source: Sci Rep. 2021 Dec 15;11:24067. doi: 10.1038/s41598-021-02527-1 (PMC8674255; doi:10.1038/s41598-021-02527-1)
Supplement: Supplementary file 1 — Supplementary Information 1. [file 41598_2021_2527_MOESM1_ESM.docx]

**ONLINE RESOURCE 2**

**Journal of Neuro-Oncology**

**Safety and tolerability of asunercept plus standard radiotherapy / temozolomide in Asian patients with newly-diagnosed glioblastoma: A Phase I study**

Kuo-Chen Wei^1,2^, Peng-Wei Hsu^1^, Hong-Chieh Tsai^1,3^, Ya-Jui Lin^1^, Ko-Ting Chen^1^, Cheng-Hong Toh^4^, Hui-Lin Huang^5^, Shih-Ming Jung^6^, Chen-Kan Tseng^7^, Yu-Xiong Ke^8^

**Corresponding author**

Prof. Kuo-Chen Wei. Department of Neurosurgery, Chang Gung Memorial Hospital, Linkou, 5 Fuxing St., Guishan Dist., Taoyuan 33305, Taiwan. Email: [kuochenwei@cgmh.org.tw](mailto:kuochenwei@cgmh.org.tw)

**Supplementary Table 1. CD95L promoter methylation in 70 clinical samples**

DNA extraction was performed on formalin-fixed parrafin embedded tissue samples using a AmoyDx® FFPE DNA extraction kit (Cat. No. ADx-FF01) (centrifugal column type) with the ADx-ARMS platform, following the manufacturer’s instructions. The DNA methylation transformation step was performed according to the Qiagen Bisulfite Kit instruction manual (Cat. No. 59824). Methylation was detected using a real-time fluorescent quantitative PCR instrument (Shanghai Hongshi-Slan series). Three repeat measurements of methylation were made and the average is presented.

|  | **CpG2 methylation, %** |
| --- | --- |
| Median | 52.0 |
| Mean value for each sample^a^ |  |
| 1 | 51.93 |
| 2 | 53.09 |
| 3 | 51.56 |
| 4 | 34.88 |
| 5 | 42.06 |
| 6 | 34.67 |
| 7 | 34.67 |
| 8 | 48.11 |
| 9 | 46.95 |
| 10 | 39.3 |
| 11 | 44.1 |
| 12 | 58.92 |
| 13 | 52.07 |
| 14 | 53.27 |
| 15 | 55.52 |
| 16 | 57.82 |
| 17 | 52.87 |
| 18 | 51.82 |
| 19 | 52.71 |
| 20 | 58.22 |
| 21 | 44.94 |
| 22 | 60.28 |
| 23 | 57.28 |
| 24 | 50.89 |
| 25 | 44.47 |
| 26 | 51.08 |
| 27 | 64.16 |
| 28 | 55.16 |
| 29 | 49.37 |
| 30 | 51.88 |
| 31 | 49.67 |
| 32 | 28.12 |
| 33 | 56.67 |
| 34 | 53.56 |
| 35 | 51.3 |
| 36 | 62.65 |
| 37 | 60.76 |
| 38 | 39.3 |
| 39 | 55.87 |
| 40 | 57.78 |
| 41 | 44.28 |
| 42 | 52.9 |
| 43 | 55.11 |
| 44 | 59.66 |
| 45 | 59.46 |
| 46 | 33 |
| 47 | 57.34 |
| 48 | 59.19 |
| 49 | 41.95 |
| 50 | 61.33 |
| 51 | 50.53 |
| 52 | 54.35 |
| 53 | 51.64 |
| 54 | 47.89 |
| 55 | 35.55 |
| 56 | 54.83 |
| 57 | 39.63 |
| 58 | 57.52 |
| 59 | 25.71 |
| 60 | 58.37 |
| 61 | 59.45 |
| 62 | 52.03 |
| 63 | 45.66 |
| 64 | 35.86 |
| 65 | 22.32 |
| 66 | 48.9 |
| 67 | 56.18 |
| 68 | 55.06 |
| 69 | 51.53 |
| 70 | 43.92 |

^a^Mean value of three duplicate measurements

**Supplementary Table 2. Study medication exposure**

| **Study period** | **Cohort 1, Asunercept 200 mg/week (n=3)**) | **Cohort 2, Asunercept 400 mg/week (n=7)** |
| --- | --- | --- |
| Overall study period  Number of patients  Median dose (min, max), mg | 3  2000 | 7  19,200 |
| Concomitant TMZ / RT period  Number of patients  Median dose (min, max), mg | 3  1,200 | 7  2,400 |
| Maintenance period  Number of patients  Median dose (min, max), mg | 3  800 | 5  17,200 |

**Supplementary Table 3. Individual CD95L promoter methylation and CD95L expression data (n=10)**

| Cohort | Weekly dose, mg | CD95L promoter methylation, % | CD95L expression | | PFS, months | DoT, months | Overall response^a^ |
| --- | --- | --- | --- | --- | --- | --- | --- |
| 1 | 200 | 3.65 | 2+ | Positive | 2.33 | 2.33 | PD |
| 1 | 200 | 42.61 | 3+ | Positive | 2.37 | 2.37 | PD |
| 1 | 200 | 46.42 | 2+ | Positive | 6.01 | 6.01 | PD |
| 2 | 400 | 36.54 | 1+ | Negative | 4.01 | 4.01 | PD |
| 2 | 400 | 42.08 | 3+ | Positive | NA | 11.53 | PR |
| 2 | 400 | 49.87 | 3+ | Positive | NA | 11.53 | SD |
| 2 | 400 | 57.2 | 3+ | Positive | NA | 17.12 | SD |
| 2 | 400 | 57.57 | 3+ | Positive | 2.33 | 2.33 | PD |
| 2 | 400 | 60.11 | 2+ | Positive | 2.30 | 2.30 | PD |
| 2 | 400 | 67.12 | 3+ | Positive | NA | 13.27 | SD |

^a^Tumor response evaluated using the Response Assessment in Neuro-Oncology criteria.

DoT, duration of treatment; NA, not evaluable; PD, progressive disease; PR, partial response; SD, stable disease.
