# Supplementary material for: Safety and tolerability of asunercept plus standard radiotherapy/temozolomide in Asian patients with newly-diagnosed glioblastoma: a phase I study
Source: Sci Rep. 2021 Dec 15;11:24067. doi: 10.1038/s41598-021-02527-1 (PMC8674255; doi:10.1038/s41598-021-02527-1)
Supplement: Supplementary file 2 — Supplementary Information 2. [file 41598_2021_2527_MOESM2_ESM.docx]

**ONLINE RESOURCE 1**

**Journal of Neuro-Oncology**

**Safety and tolerability of asunercept plus standard radiotherapy / temozolomide in Asian patients with newly-diagnosed glioblastoma: A Phase I study**

Kuo-Chen Wei^1,2^, Peng-Wei Hsu^1^, Hong-Chieh Tsai^1,3^, Ya-Jui Lin^1^, Ko-Ting Chen^1^, Cheng-Hong Toh^4^, Hui-Lin Huang^5^, Shih-Ming Jung^6^, Chen-Kan Tseng^7^, Yu-Xiong Ke^8^

**Corresponding author**

Prof. Kuo-Chen Wei. Department of Neurosurgery, Chang Gung Memorial Hospital, Linkou, 5 Fuxing St., Guishan Dist., Taoyuan 33305, Taiwan. Email: [kuochenwei@cgmh.org.tw](mailto:kuochenwei@cgmh.org.tw)

**Patient enrollment criteria**

The study included adult Taiwanese patients (aged ≥20 to <75 years) with newly diagnosed and histologically confirmed glioblastoma, life expectancy ≥6 months, and a tumor that was surgically accessible with available tissue samples. Eligible patients also had a Karnofsky Performance Scale (KPS) Index score ≥60 prior to treatment and had not received prior therapy for brain tumors. Laboratory criteria for enrolment included an absolute neutrophil count ≥1.5×10^9^/L, a platelet count ≥100×10^9^/L, hemoglobin ≥10 g/dL, creatinine ≤1.25 × upper limit of normal (ULN), and adequate hepatic function (total bilirubin ≤1.5 × ULN, aspartate aminotransferase [AST] and alanine aminotransferase [ALT] ≤2.5 × ULN). In addition, patients were required to have undergone baseline magnetic resonance imaging (MRI) within 2 days of surgery. Key exclusion criteria were prior receipt of any chemotherapy, immunotherapy or radiotherapy to the brain, any concurrent malignancy other than basal cell carcinoma or carcinoma *in situ* of the cervix, low-grade astrocytoma, HIV infection, and positivity for anti-hepatitis C virus antibody. Patients positive for hepatitis B virus surface antigen who had received any related treatment within the past 6 months were also excluded.

**Patient assignment algorithm**

If none of the first three patients enrolled to Cohort 1 (asunercept 200 mg/week) experienced dose-limiting toxicity (DLT), dose escalation to 400 mg/week was permitted. However, if one of the three patients experienced a DLT, then Cohort 1 was expanded to six evaluable patients and dose escalation was only permitted if one of the total cohort of six patients experienced a DLT, if two or more patients in a group of up to six patients experienced a DLT (irrespective of the number of patients enrolled) then the dose was considered not tolerated and recruitment to the cohort and dose escalation were ceased. For Cohort 2, if one or fewer of the first three patients enrolled experienced a DLT, the cohort was expanded to include six evaluable patients. Subsequently, if one or fewer of the six patients experienced a DLT, an additional three patients were enrolled, giving a total of nine patients for further DLT assessment. Enrolment of the final three patients to Cohort 2 was also dependent on a review of observed DLTs. If two or more patients in the dose group of three to six patients experienced DLTs, dose escalation would be stopped, and three patients would enter the next lower dose level (if applicable) unless six or more patients were treated previously at that dose. The final approval of dose escalation was given by the safety monitoring committee (SMC) following the review of safety data from the 6 weeks of concurrent treatment with asunercept in combination with radiotherapy concomitant with temozolomide (RT/TMZ).

**Schedule of assessments**

Physical examinations, measurement of vital signs, KPS, and laboratory safety were assessed during screening, at baseline, during weekly assessments from Weeks 2–6, at the Week 7 end of radiotherapy assessment, every 4 weeks following completion of radiotherapy, at the end of study treatment, at the 28-day post-treatment follow-up, and then every 8 weeks subsequently until disease progression or withdrawal.

**CpG2 methylation detection**

DNA extraction was performed using a AmoyDx® FFPE DNA extraction kit (Cat. No. ADx-FF01) (centrifugal column type) with the ADx-ARMS platform, following the manufacturer’s instructions. The DNA methylation transformation step was performed according to the Qiagen Bisulfite Kit instruction manual (Cat. No. 59824). Methylation was detected using a real-time fluorescent quantitative PCR instrument (Shanghai Hongshi-Slan series).
